# Vitexin compound 1, a novel extraction from a Chinese herb, suppresses melanoma cell growth through DNA damage by increasing ROS levels

**DOI:** 10.1186/s13046-018-0897-x

**Published:** 2018-11-06

**Authors:** Nian Liu, Kuan Song Wang, Min Qi, Ying Jun Zhou, Guang Yao Zeng, Juan Tao, Jian Da Zhou, Jiang Lin Zhang, Xiang Chen, Cong Peng

**Affiliations:** 1 Department of Dermatology, Xiangya Hospital, Central South University, Changsha, Hunan China; 2 Hunan Key Laboratory of Skin Cancer and Psoriasis, Changsha, Hunan China; 3 Department of Pathology, Xiangya Hospital, Central South University, Changsha, Hunan China; 4 Department of Pathology, School of Basic Medical Sciences, Central South University, Changsha, Hunan China; 5 Department of Plastic and Cosmetic Surgery, XiangYa Hospital, Central South University, Changsha, Hunan China; 6 School of Pharmaceutical Science,Central, South University, Changsha, Hunan China; 7 Department of Dermatology, Affiliated Union Hospital, Tongji Medical College, Huazhong University of Science and Technology, Wuhan, China; 8 Department of Plastic Surgery of Third Xiangya Hospital, Central South University, Changsha, China

**Keywords:** Vitexin compound 1, Melanoma, ROS, DNA damage

## Abstract

**Background:**

*Vitex negundo* L (Verbenaceae) is an aromatic shrub that is abundant in Asian countries. A series of compounds from *Vitex negundo* have been used in traditional Chinese medicine for the treatment of various diseases. Cutaneous melanoma is one of the most aggressive malignancies. A significant feature of melanoma is its resistance to traditional chemotherapy and radiotherapy; therefore, there is an urgent need to develop novel treatments for melanoma.

**Methods:**

We first examined the effects of VB1 (vitexin compound 1) on cell viability by CCK-8 (cell counting kit) and Colony Formation Assay; And then, we analyzed the apoptosis and cell cycle by flow cytometry, verified apoptosis by Immunoblotting. The in vivo effect of VB1 was evaluated in xenograft mouse model. Potential mechanisms of VB1’s antitumor effects were explored by RNA sequencing and the key differential expression genes were validated by real-time quantitative PCR. Finally, the intracellular reactive oxygen species (ROS) level was detected by flow cytometry, and the DNA damage was revealed by Immunofluorescence and Immunoblotting.

**Results:**

In this study, we show that VB1, which is a compound purified from the seed of the Chinese herb *Vitex negundo*, blocks melanoma cells growth in vitro and in vivo, arrests the cell cycle in G2/M phase and induces apoptosis in melanoma cell lines, whereas the effects are not significantly observed in normal cells. To study the details of VB1, we analyzed the alteration of gene expression profiles after treatment with VB1 in melanoma cells. The findings showed that VB1 can affect various pathways, including p53, apoptosis and the cell cycle pathway, in a variety of melanoma cell lines. Furthermore, we confirmed that VB1 restored the P53 pathway protein level, and then we demonstrated that VB1 significantly induced the accumulation of ROS, which resulted in DNA damage in melanoma cell lines. Interestingly, our results showed that VB1 also increased the ROS levels in BRAFi (BRAF inhibitor)-resistant melanoma cells, leading to DNA cytotoxicity, which caused G2/M phase arrest and apoptosis.

**Conclusions:**

Taken together, our findings indicate that vitexin compound 1 might be a promising therapeutic Chinese medicine for melanoma treatment regardless of BRAFi resistance.

**Electronic supplementary material:**

The online version of this article (10.1186/s13046-018-0897-x) contains supplementary material, which is available to authorized users.

## Background

Melanoma is a malignant tumor with a progressively increasing incidence and a poor prognosis around the world [1]. Cutaneous melanoma is the most common subtype of melanoma; it accounts for more than 90% of melanomas [2, 3] and exhibits poor response to both traditional chemotherapy and radiotherapy [4]. Recently, based on understanding the details of this disease, breakthroughs have been made in the treatment of advanced melanoma [5]. Key genomic mutation genes, including *BRAF* (35–60%) and *NRAS* (15–20%), have been identified, which has led to the development of targeted therapeutic treatments for advanced melanoma [6, 7]. Targeted inhibitors such as vemurafenib (BRAF inhibitors, BRAFi), trametinib (MEK inhibitor, MEKi) and the combination of BRAFi+MEKi have been approved by the FDA for advanced melanoma patients carrying the V600E residue of the BRAF protein [8]. BRAF inhibitors, alone and in combination with MEK inhibitors, significantly reduce the tumor burden and improve the progression-free survival and response rates among advanced melanoma patients. However, the benefits of BRAFi monotherapy are only temporary; after 6~ 7 months, most patients receiving monotherapy develop drug resistance [9, 10]. Although the FDA has also approved several immunotherapies for advanced melanoma patients [11], the response rates are generally lower, and the severe side effects of immunotherapy are fatal for patients [12]. Therefore, there is an urgent need to develop novel drugs with low toxicity for the treatment of melanoma patients.

*Vitex negundo* L (Verbenaceae) is an aromatic shrub that is abundant in Asian countries [13]. Recently, a series of compounds from *Vitex negundo* have been used in traditional Chinese medicine for the treatment of various diseases [14–17]. For example, the compounds vitexin and isovitexin extracted from *Vitex negundo* were demonstrated to prevent myocardial ischemia–reperfusion injury and to exhibit anti-inflammatory or antioxidant properties [18]. EVn-50, which is another mixture of compounds from *Vitex negundo* seeds, shows broad antitumor activity for colon cancer, breast cancer, ovarian cancer, pancreatic cancer and breast cancer [14]. Purified VB1 is the most abundant vitexin compound in the EVn-50 mixture, and it has been found to inhibit growth and angiogenesis through suppression of the AKT/FOXO3 pathway in hepatocellular carcinoma [19], to suppress the growth of choriocarcinoma by inhibiting mTOR signaling [20] and to exert a broad-spectrum cytotoxic effect by arresting cancer cells at G2/M phase cell cycle in many cancers [21]. However, no studies have addressed the effects of VB1 on melanoma. In addition, there is no comprehensive explanation of the molecular mechanism of VB1.

In this study, we found that VB1, which is the most abundant vitexin compound in the EVn-50 mixture of compounds, inhibits the growth of melanoma cells in vitro and in vivo by inducing DNA damage by regulating ROS accumulation. Interestingly, VB1 also blocks the growth of BRAFi-resistant melanoma cells regardless of resistance, which indicates that VB1 is a promising medicine for melanoma treatment.

## Methods

### Chemical

**Fig. 1:**
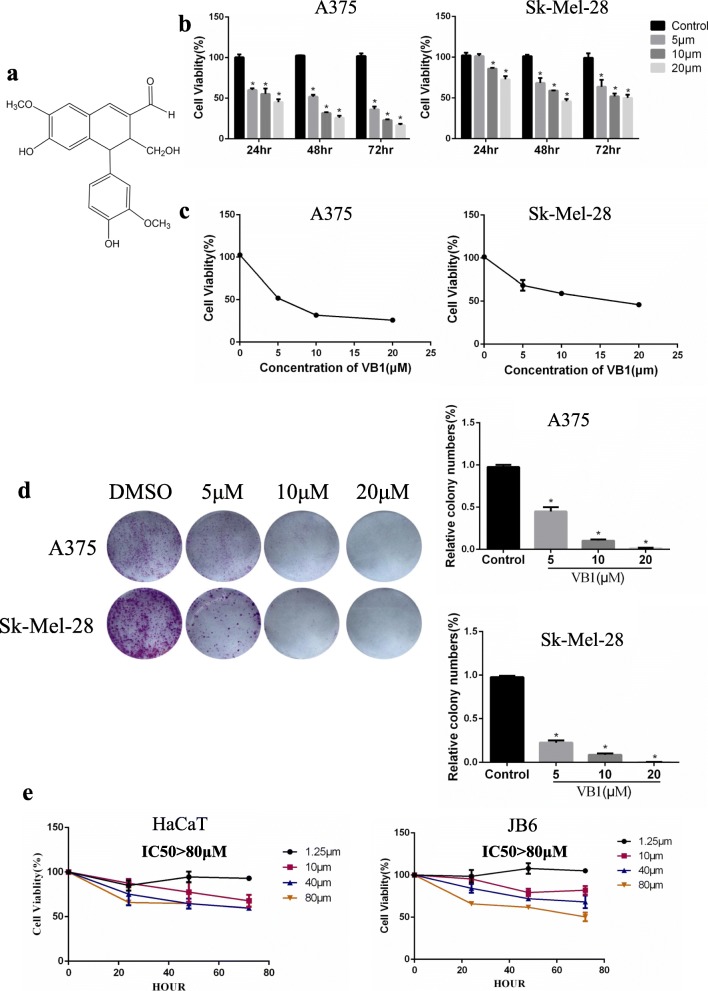
VB1 blocks the proliferation of melanoma cells. **a** Chemical structure of VB1. **b** A375 and Sk-Mel-28 cells were prepared in 96-well plates. The cells were treated with VB1 for various times and dosages as indicated, and cell viability was tested by CCK-8 as described in the Methods. The data from multiple experiments are expressed as the mean (*n* = 6) ± S.D. Significant differences were evaluated using one-way ANOVA, and an asterisk (*) indicates a significant difference (*p* < 0.05). **c** The IC50 values of VB1 in A375 and Sk-Mel-28 were automatically calculated by GraphPad Prism software as described in the *Materials and Methods*. **d** A375 and Sk-Mel-28 cells were seeded into 6-well plates, and then treated with various dosages of VB1 as indicated for 24 h. After 10–14 days, the number of colonies was assessed and quantified by crystal violet staining as described in the Methods. The data represent the mean (*n* = 4) ± SD of each group, and an asterisk (*) indicates a significant difference evaluated using one-way ANOVA (*p* < 0.05). **e** Nontumorigenic HaCaT and JB6 cells were treated with VB1 for different times and dosages as indicated. Cell viability was determined by the CCK-8 assay. The data from multiple experiments are expressed as the mean (*n* = 6) ± S.D. Significant differences were evaluated using one-way ANOVA, and an asterisk (*) indicates a significant difference (*p* < 0.05)

VB1 (vitexin compound-1, 6-hydroxy-4-(4-hydroxy-3-methoxyphenyl)-3-hydroxymethyl-7-methoxy-3,4-dihydro-2-naphthaldehyde), which is a compound purified from the seed of the Chinese herb *Vitex negundo*, was extracted and isolated by our team member from the College of Pharmacy of Central South University (Changsha, China) (Fig. 1a). A VB1 stock solution was prepared by dissolving the compound in dimethyl sulfoxide (DMSO) and diluting it with pure water to a concentration of 2.5 mmol/L. The VB1 was packed and stored at 4 °C. The final concentration of DMSO in each sample was less than 0.02% (vol/vol).

### Cell lines and culture

Human malignant melanoma cell lines A375, Sk-Mel-5 and Sk-Mel-28 (American Type Culture Collection, USA) and vemurafenib-resistant A375(called RA) was generated as described in previous study [22]), were used in this study. The cells were grown in Dulbecco’s modified Eagle’s medium (BI, Israel) supplemented with 10% fetal bovine serum (BI, Israel) at 37 °C and 5% CO_2_. The RA cell line was used for drug resistance at 2 micro-moles per milliliter of vemurafenib, and the drug was removed one week before use.

### Cytotoxicity assay (CCK-8)

Cells were seeded into 96-well plates (2 × 10^3^ cells per well) to allow attachment and incubated overnight at 37 °C in media containing 10% FBS. This was followed by exposure to various concentrations of VB1 or DMSO (control) for 24, 48 and 72 h. The cell viability (%) was determined by CCK-8 assay (Selleck, USA) according to the manufacturer’s instructions. The fluorescence of each plate was measured using a spectrophotometer at an emission of 450 nm (Beckman, USA). Each sample had 6 replicates. The cells in the control group were treated with equal amounts of DMSO. The half-maximal inhibitory concentration (IC50) values were calculated for 48 h using the GraphPad Prism software.

### Colony formation assay

Cells were seeded into 6-well plates (1–1.5 × 10^3^ cells per well) to allow attachment and incubated overnight at 37 °C in media containing 10% FBS followed by exposure to various concentrations of VB1 or DMSO (control). After 24 h, the drug-containing medium was removed and replaced with complete growth medium. The medium was then replaced every 3 days for 14 days until visible colonies formed. The colonies were simultaneously fixed with 4% paraformaldehyde and stained with 0.5% crystal violet. The dishes were washed with PBS. Visible colonies containing no less than 50 cells were counted.

### Cell apoptosis and cell cycle assay

Cells were seeded into 6-well plates (3 × 10^5^ cells per well) to allow attachment, incubated overnight at 37 °C in media containing 10% FBS and treated with various concentrations of VB1 or DMSO (control). For the cell cycle assays, the cells were harvested by trypsinization after 48 h. The collected cells were washed with cold PBS and then fixed overnight in ice-cold 70% ethanol. The next day, the collected cells were incubated with propidium iodide (PI) staining (Becton, Dickinson and Company, USA) in the dark at room temperature according to the manufacturer’s instructions. The cell cycle was measured by flow cytometry and analyzed using ModFit software. For the cell apoptosis assays, the cells were harvested by trypsinization without EDTA after 48 h. The collected cells were washed with cold PBS and incubated with Annexin V/propidium iodide staining (Becton, Dickinson and Company, USA) according to the manufacturer’s instructions. Cell apoptosis was detected by flow cytometry and analyzed using the FlowJo software.

### Immunoblotting

The cells were lysed with RIPA (Radio-Immunoprecipitation Assay) buffer (DingGuo, China) containing protease and phosphatase inhibitors (Selleck, USA). The total protein concentration in the cell lysate was determined with a BCA protein assay kit (Beyotime, China). Proteins were separated by 8–12% SDS-polyacrylamide gel electrophoresis (SDS-PAGE), transferred to polyvinylidene fluoride membranes (Millipore, USA) and incubated with primary and secondary antibodies: PARP (1:1000, CST), BCL2 (1:1000, Protech), BAX (1:1000, Protech), γH2AX (1:1000, CST), P-ATM (1:1000, CST), P-ATR (1:1000, CST), P-CHK2 (1:1000, CST), P53 (1:1000, Santa), P21 (1:1000, CST), ATM (1:1000, Protech), ATR (1:1000, Protech), CHK2 (1:1000, Protech), GAPDH (1:3000, Protech), β-actin (1:3000, Protech), and α-tubulin (1:2000, Protech). The results were imaged using a gel image analysis system (Bio-Rad, USA) according to the manufacturer’s instructions.

### RNA-sequencing

The cDNA library construction, library purification and transcriptome sequencing were implemented according to the Wuhan Huada Sequencing Company’s instructions.

### Quantitative real-time PCR analysis

Total RNA was prepared according to the manufacturer’s instructions. cDNA was synthesized by the SuperScript III First-Strand Synthesis System for reverse transcription PCR (Invitrogen, USA). qRT-PCR was performed using SYBR Green qPCR mix (Toyobo, Japan). All of the PCR primers used in this study are listed in Table 1. The qRT-PCR assays and data collection were performed on a 7500 real-time PCR system (Applied Biosystems, USA). Data were analyzed by using 2^-△△CT^ values.

**Table 1:** The primers used in the PCR reaction and annealing

Gene name	Sequence (5’to3’)	Direction
CDKN1A	AGCGACCTTCCTCATCCACC	Forward
CDKN1A	AAGACAACTACTCCCAGCCCCATA	Reverse
CCNE2	ATCTCCTGGCTAAATCTCTTTCTCC	Forward
CCNE2	ACTGGAACTCTAATGAATCAATGGC	Reverse
CCNA2	TTTAGCACTCTACACAGTCACGGGA	Forward
CCNA2	GGTGAAGGTCCATGAGACAAGGC	Reverse
CDK6	AGAGCAAGATAATAAAGGAGATGGG	Forward
CDK6	CATGTGAGACTTTGAGTAGACCTGA	Reverse
MCM6	GCTGTCGCACTGTAATCCTCC	Forward
MCM6	ATTGATCGTGTCTATTCCCTCG	Reverse
BBC3	TCTCCTCTCGGTGCTCCTTCACT	Forward
BBC3	ACGTTTGGCTCATTTGCTCTTCA	Reverse
GADD45A	CTCAAGCAGTTACTCCCTACAC	Forward
GADD45A	CTTCTTCATTTTCACCTCTTTCCA	Reverse
CDK1	TAGTCTGGTCTTTCTTTGGCTG	Forward
CDK1	GTTCAAAACTGGAATAAAACACCTA	Reverse

### Measurement of ROS

Cells were seeded into 6-well plates (3 × 10^5^ cells per well) to allow attachment, incubated overnight at 37 °C in media containing 10% FBS and treated with various concentrations of VB1 or DMSO (control) for 0–12 h. The pretreated cells were then loaded with DCFH-DA (Solarbio, China) in DMEM at 37 °C and incubated for 20 min according to the manufacturer’s instructions. Excess DCFH-DA was removed by washing with DMEM. The ROS levels were measured by flow cytometry and analyzed using the FlowJo software. In a different experiment, the cells were pretreated with 5 mmol/L N-acetylcysteine (NAC) (Beyotime, China) for 1 h before exposure to VB1 and cultured for another 6 h. The ROS levels were measured by flow cytometry and analyzed using the FlowJo software.

### Immunofluorescence

Cells were seeded into 6-well plates (3 × 10^5^ cells per well) to allow attachment, incubated overnight at 37 °C in media containing 10% FBS and treated with various concentrations of VB1 or DMSO (control) for 24 and 48 h. The cells were then fixed with 4% paraformaldehyde and permeabilized in 0.5% Triton X-100. After blocking with 5% bovine serum albumin, the samples were incubated with γH2AX antibody and secondary antibody. The cells were counterstained with DAPI and visualized by fluorescence microscopy.

### Immunohistochemistry

Formalin-fixed, paraffin-embedded tumor sections were baked at 65 °C, deparaffinized in turpentine, rehydrated through a series of graded alcohol, and immersed in hydrogen peroxide to block endogenous peroxidase activity. Antigen retrieval was applied by heat treatment in a pressure cooker in a citrate buffer (pH 6.0). Sections were then blocked for nonspecific binding by incubation in normal goat serum at 37 °C. Subsequently, the slides were incubated with a primary antibody of Ki67 (1:400, Abcam), γH2AX (1:200, CST), P53 (1:200, Santa) at 4 °C overnight. The next day, the sections were incubated with the secondary antibody at 37 °C. The slides were then added to a horseradish peroxidase-conjugated streptomycin working solution and stained with DAB reaction.

### Xenograft tumor model

A total of 2 × 10^6^ Sk-Mel-5 melanoma cells were injected subcutaneously into the right flank of 4- to 5-week-old athymic BALB/c female nude mice (nu/nu). When the tumors reached 50 mm^3^ or larger, the tumor-bearing mice were randomized for intraperitoneal injection of 40 or 80 mg/kg VB1 or 0.5% carboxymethyl cellulose sodium (CMC, control) twice every other day for 2–3 weeks, with 7 mice in each group. The tumor size was measured using a caliper every other day, and the tumor volume was calculated with the formula V = 1/2(length×width^2^). When the tumors reached 1000 mm^3^, the tumor-bearing mice were sacrificed for the histological analysis.

### Statistical analysis

Student’s t-tests and one- or two-way ANOVA tests were conducted to analyze the data using the GraphPad Prism software (version 6.01). The quantified data are presented as the mean ± SEM. Differences were considered to be significant when *P* < 0.05.

## Results

### VB1 suppressed the proliferation of melanoma cells

We examined the effects of VB1 on cell viability in multiple melanoma cell lines by CCK-8 assay. As shown in Fig. 1b, c and Additional file 1: Figure S1A (upper panel), VB1 reduces cell viability in a dosage- and time-dependent manner. The IC50 values of VB1 for A375, SK-MEL-28 and SK-MEL-5 were 5 μM, 15 μM and 12 μM, respectively (Fig. 1c and Additional file 1: Figure S1A (lower panel)). To further evaluate the inhibitory efficacy of VB1, we treated melanoma cell lines with VB1 for 24 h and then assessed cellular colony formation and growth in the plates. The results showed that colony formation was suppressed by VB1 in a dosage-dependent manner (Fig. 1d and Additional file 1: Figure S1B), In addition, to determine the cellular toxicity of VB1 in normal cells, we exposed the immortalized nontumorigenic mouse skin epidermal cell line (JB6) and the human skin keratinocytes cell line (HaCat) to higher dosages of VB1. The results showed that the IC50 values of VB1 for the two cell lines were over 80 μM (Fig. 1e), which is much greater than its efficacious concentration in melanoma cells. These results suggest that the cytotoxicity of VB1 was selective to melanoma cells.

### VB1 induced apoptosis and G2/M cell cycle arrest in melanoma cells

**Fig. 2:**
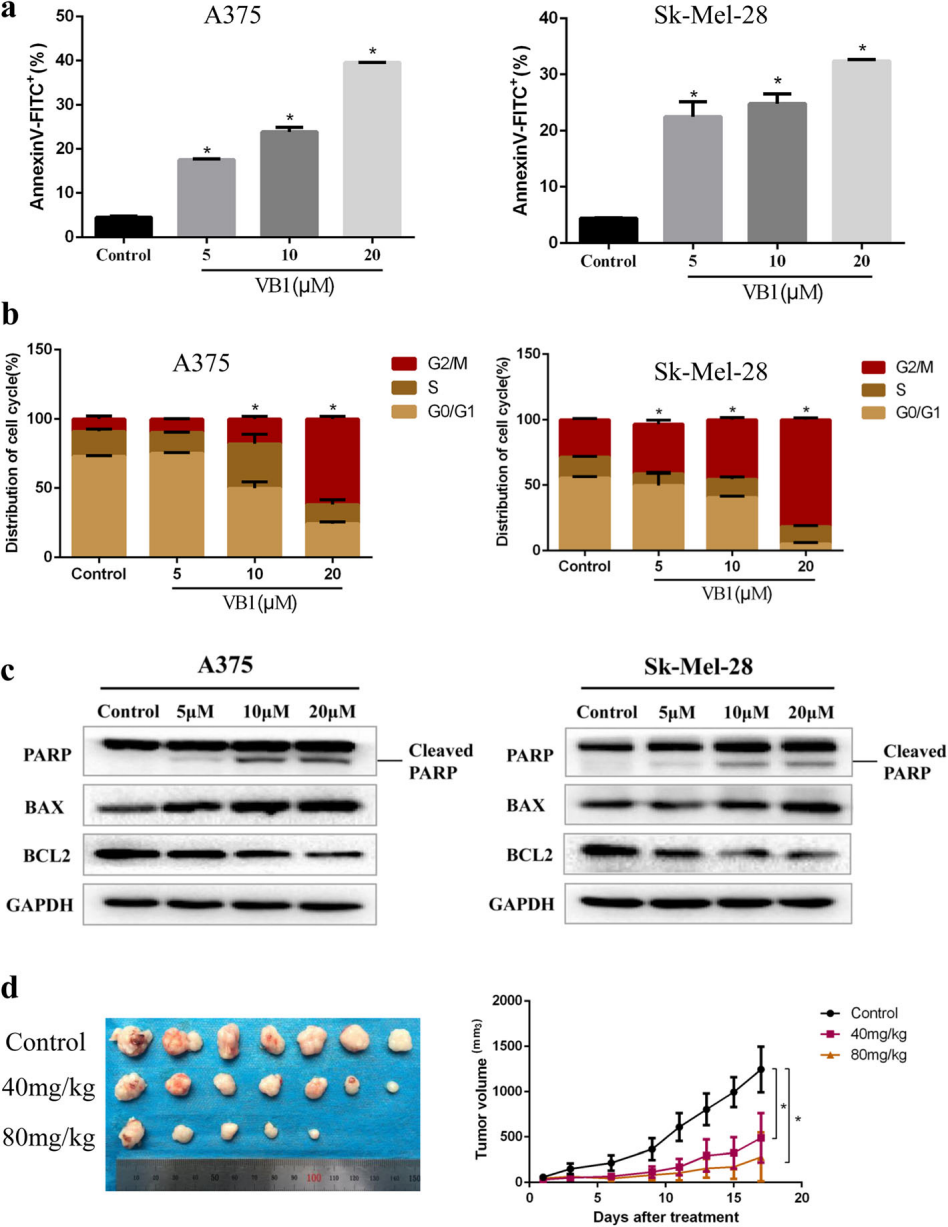
VB1 treatment induces apoptosis and cell cycle arrest in melanoma cells. **a** A375 and Sk-Mel-28 cells were treated with various dosages of VB1 for 48 h, and the extent of apoptosis was determined by flow cytometry with Annexin V and PI double staining as described in the Methods. The results represent the mean (*n* = 4) ± SD of each group, and an asterisk (*) indicates a significant difference using one-way ANOVA (*p* < 0.05). **b** A375 and Sk-Mel-28 cells were treated with various dosages of VB1 for 48 h. The cell cycle distribution was detected by flow cytometry as described in the Methods. The results represent the mean (*n* = 4) ± SD of each group, and an asterisk (*) indicates a significant difference using one-way ANOVA (*p* < 0.05). **c** A375 and Sk-Mel-28 cells treated with various dosages of VB1 were lysed, and western blotting was then performed for the indicated antibodies. **d** Sk-Mel-5 melanoma cells (2 × 10^6^ cells/0.15 mL) were xenografted into nude mice. When the tumors reached approximately 50 mm^3^, the tumor-bearing mice were randomized for intraperitoneal injection of 40 or 80 mg/kg of VB1 twice every other day for 2–3 weeks as described in the Methods. The tumor growth and body weight were measured twice per week. The results are shown as the mean tumor volume ± SD, and an asterisk (*) indicates a significant difference (*p* < 0.05 one-way ANOVA)

To clarify the details of VB1-induced cytotoxicity in melanoma cells, we analyzed the apoptosis and cell cycle in A375, Sk-Mel-28 and Sk-Mel-5 cells and found that VB1 could significantly induce apoptosis and G2/M cell cycle arrest in a dosage-dependent manner. At a low dose of 5 μM, VB1 induced 17.48%, 16.47% and 17.03% apoptosis in A375, Sk-Mel-28 and Sk-Mel-5 cells, respectively. At a dose of 20 μM, the apoptosis increased to 39.6%, 32.4% and 39.1%, respectively (Fig. 2a and Additional file 1: Figure S1C). In addition, 5 μM VB1 caused a change in the cell cycle distribution in the G2/M phase arrest, whereas 20 μM VB1 significantly induced arrest at G2/M phase by decreasing the distribution of G0/G1 phase (Fig. 2b and Additional file 1: Figure S1D). Next, we examined the expression of apoptotic markers during VB1 treatment. The results showed that VB1 induced cleavage of PARP and upregulated BAX expression, whereas BCL2 expression was downregulated after VB1 treatment in different melanoma cell lines (Fig. 2c and Additional file 1: Figure S1E). To examine the effect of VB1 on cancer cell growth in vivo, a xenograft study was performed in nude mice, as shown in Fig. 2d. The results were consistent with the in vitro results; VB1 completely attenuated the growth of the xenografted melanoma cells, and there was no significant change in the body weight of the tumor-bearing mice (Fig. 2d and Additional file 1: Figure S1F, G).

### Effect of VB1 on the gene expression profile and the alteration of key pathways

**Fig. 3:**
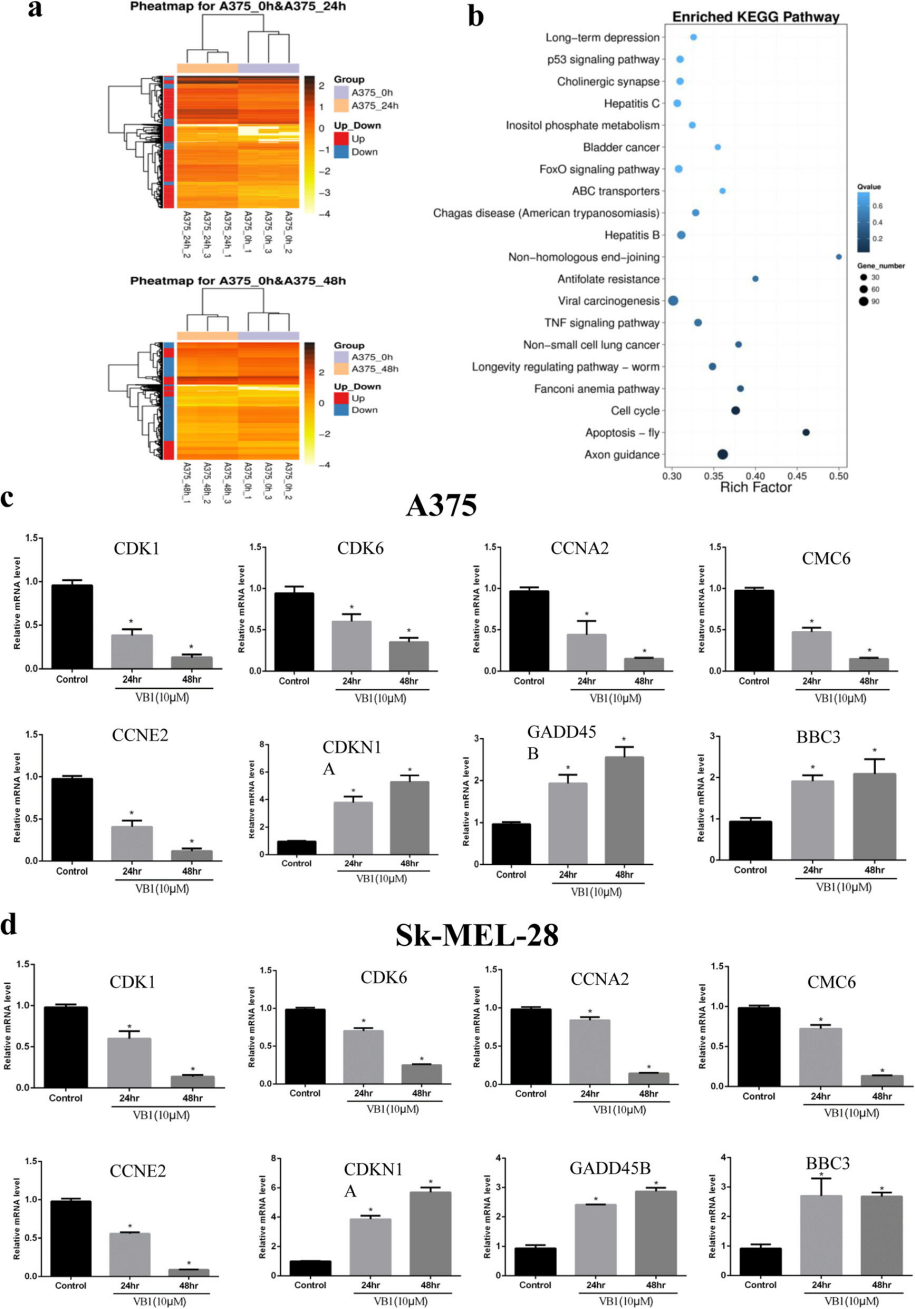
RNA-seq analyses of the effect of VB1 on the gene expression profile. **a** A375 cells were treated with 10 μM VB1 for 48 h. RNA-seq was performed as described in the Methods, and differential expression genes were analyzed using DESeq2. **b** The KEGG pathway was used to analyze the pathways related to the differential expression genes. The top 20 positively enriched pathways are shown in the bubble chart. The *x*-axis is the enrichment score, and the *y*-axis is the enriched pathways. **c** & **d** RNA was extracted from A375 (**c**) and Sk-Mel-28 (**d**) treated with VB1 as indicated, and RT-Q-PCR was then performed as described in the Methods. The data from multiple experiments are expressed as the mean (*n* = 4) ± S.D. Significant differences were evaluated using one-way ANOVA, and an asterisk (*) indicates a significant difference (*p* < 0.05)

To further study the possible mechanisms of VB1’s antitumor effects, we analyzed the global transcriptome alteration of melanoma cells after VB1 treatment in different melanoma cell lines. The RNA-seq results showed that 409 genes were upregulated and 102 genes were downregulated after VB1 treatment for 24 h, whereas 2538 genes were upregulated and 3950 genes were downregulated after 48 h of treatment (Fig. 3a). Similar results were observed in Sk-Mel-5 cells (Additional file 2: Figure S2A (left panel)). We analyzed differential expression genes by the Kyoto Encyclopedia of Genes and Genomes (KEGG) pathway. These genes are involved in multiple pathways, such as JAK-STAT, FOXO, TNF and cell cycle-related pathways (Fig. 3b and Additional file 2: Figure S2A (right panel)). We identified pivotal pathways involved in p53, DNA damage and the cell cycle, which was consistent with the effect of VB1 on G2/M arrest in different melanoma cell lines. Next, we validated the key differential expression genes by Q-RT-PCR. Consistent with the RNA-seq results, the alteration of several genes, including *P21* (*CDKN1A*), *PUMA* (*BBC3*), *CYCE* (*CCNE*), *CYCA* (*CCNA*), *CDK1*, *CDK6* and *MCM6*, were confirmed, all of which play crucial roles in cell cycle regulation (Fig. 3c, d and Additional file 2: Figure S2B).

### VB1 induced DNA damage by increasing intracellular ROS in melanoma cells

**Fig. 4:**
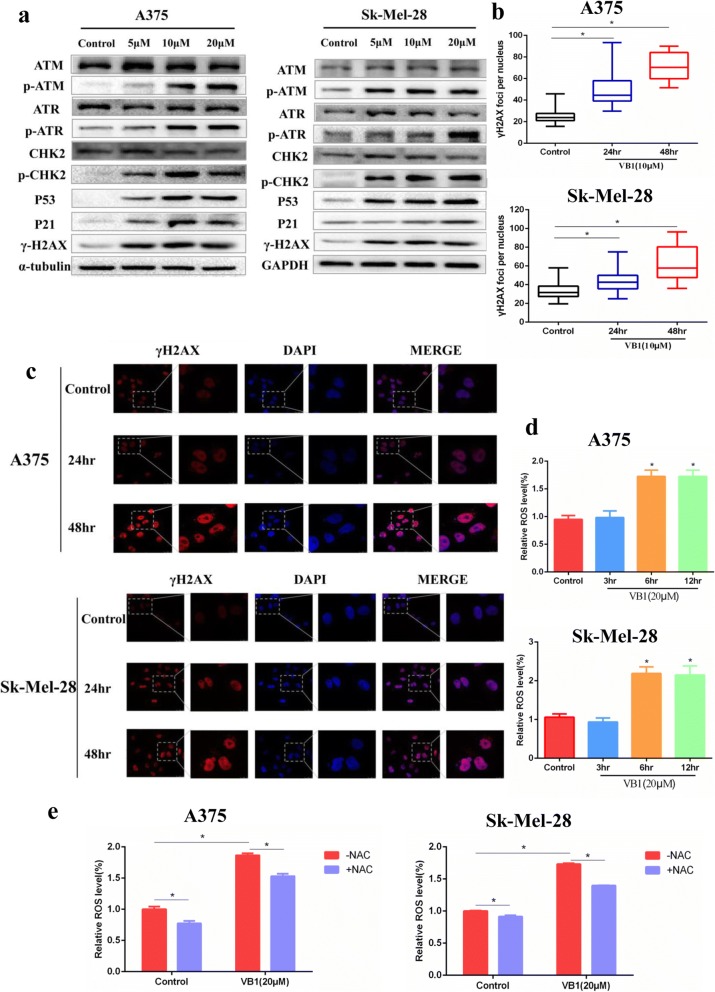
VB1 treatment induces DNA damage by increasing ROS. **a** A375 (left panel) and Sk-Mel-28 (right panel) cells were treated with 0–20 μM VB1 for 48 h, and western blotting was then performed for the indicated antibodies. **b** A375 and Sk-Mel-28 cells were treated with 10 μM VB1 for 0–48 h, and γH2AX was stained by immunofluorescence and calculated. The results represent the mean (*n* = 5) ± SD of each group, and an asterisk (*) indicates a significant difference using one-way ANOVA (*p* < 0.05). **c** A375 and Sk-Mel-28 cells were treated with 10 μM VB1 for 0–48 h, and γH2AX was stained by immunofluorescence. Representative images of staining of γH2AX. **d** A375 and Sk-Mel-28 cells were treated with 20 μM VB1 for 0–12 h. The levels of ROS were measured by DCF fluorescence with flow cytometry. The relative ROS levels were analyzed using the GraphPad Prism software (histogram). **e** A375 and Sk-Mel-28 cells were pretreated with 5 mmol/L N-acetylcysteine (NAC) for 1 h, and then, exposed to 20 μM VB1 for another 6 h. The levels of ROS were measured by DCF fluorescence with flow cytometry. The relative ROS levels were analyzed using the GraphPad Prism software (histogram). The results represent the mean (*n* = 4) ± SD of each group, and an asterisk (*) indicates a significant difference using one-way ANOVA (*p* < 0.05)

Based on the previous results, which showed that VB1 treatment induced significant apoptosis and G2/M arrest in melanoma cells, we proposed that VB1 might induce DNA toxicity and result in cellular apoptosis and G2/M cell cycle arrest. As expected, VB1 treatment significantly increased p53, P-ATM, P-ATR, P-CHK2 and γH2AX expression in a dosage-dependent manner. There were no significant changes in the expression of total ATM, ATR and CHK2 protein level (Fig. 4a and Additional file 3: Figure S3A). In addition, the accumulation of γH2AX was observed in the nucleus (Fig. 4b, c and Additional file 3: Figure S3B, C), which suggests that this compound induced DNA damage. We also examined Ki67, P53 and γH2AX expression in paraffin-embedded mice tumor tissues. The findings showed that P53 and γH2AX expression increased significantly after VB1 treatment and that the expression of Ki67 was inhibited (Additional file 3: Figure S3D), which was consistent with the results of the VB1 inhibition of xenografted melanoma cell growth (Fig. 2d) and induction of DNA damage. Oxidative damage is well-known to play dual roles in carcinogenesis and ROS-based anticancer treatment. At low or moderate levels, ROS regulate essential biological functions of cells, including proliferation, angiogenesis and tumor metastasis, whereas at higher levels, ROS affect cells by causing DNA damage and apoptosis that leads to therapeutic effects on cancer [23]. Our results showed that VB1 treatment dramatically increases intracellular ROS levels in different melanoma cell lines (Fig. 4d and Additional file 3: Figure S3E (left panel)), which could be partly impeded by antioxidant N-acetylcysteine (NAC) (Fig. 4e and Additional file 3: Figure S3E (right panel)). All of those indicates that VB1 treatment promotes the accumulation of ROS and leads to DNA damage in melanoma cells.

### VB1 attenuated BRAFi-resistant melanoma cell growth

**Fig. 5:**
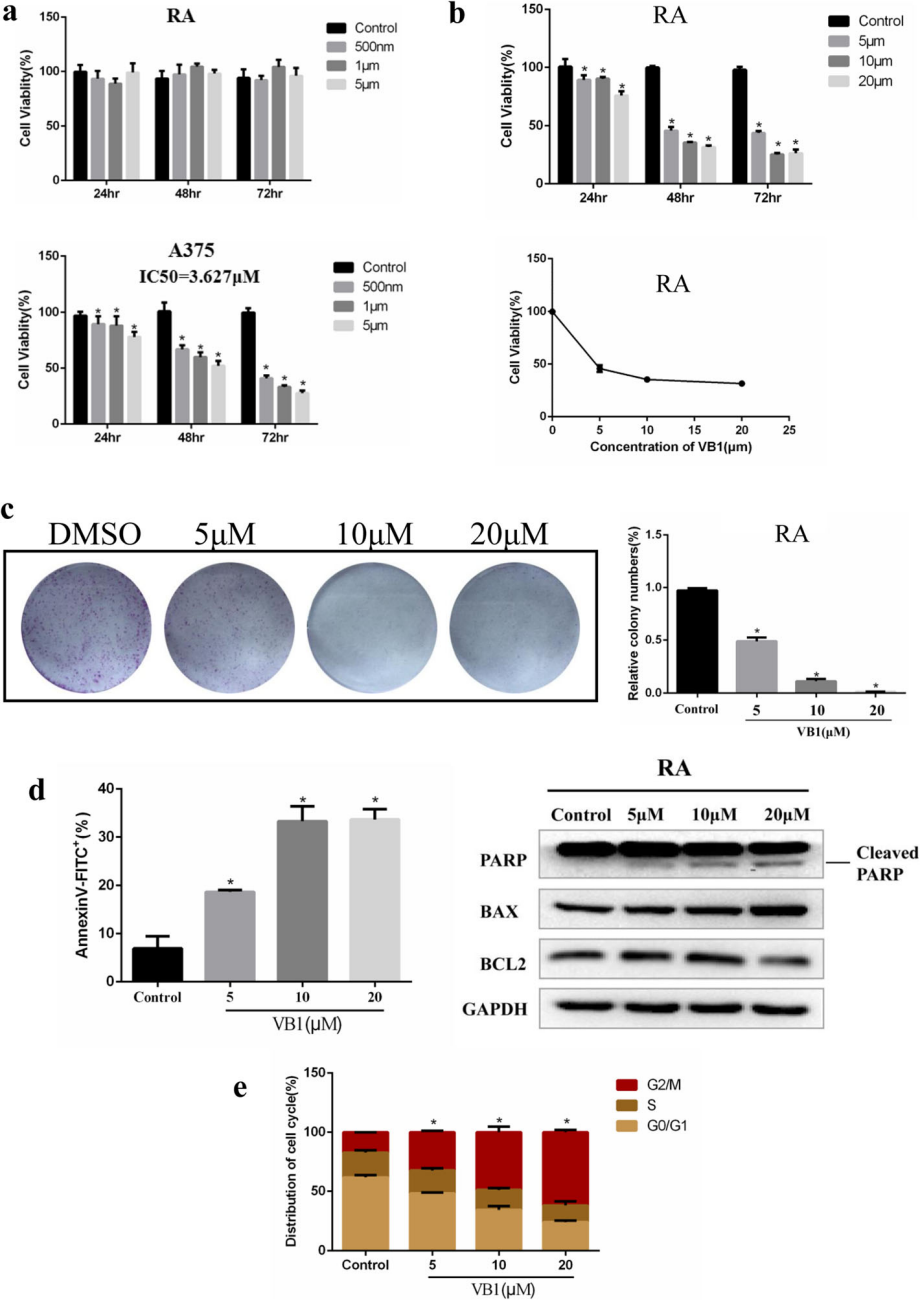
Effect of VB1 on BRAFi-resistant melanoma cells. **a** BRAFi-resistant melanoma cells (RA) from parental A375 were generated as described in the Methods. RA and parental A375 cells were seeded into 96-well plates and then treated with PLX4072. Cell viability was determined by the CCK-8 assay. The results represent the mean (*n* = 6) ± SD of each group, and an asterisk (*) indicates a significant difference using one-way ANOVA (*p* < 0.05). **b** RA cells were treated with 0–20 μM VB1 for 0–72 h. Cell viability was determined by the CCK-8 assay. The IC50 values of VB1 in the RA cells were automatically generated by the GraphPad Prism software. **c** RA cells were seeded into 6-well plates and then treated with various dosages of VB1 as indicated for 24 h. After 10–14 days, the number of colonies was assessed and quantified by crystal violet staining as described in the Methods. **d** RA cells were seeded into 6-well plates after being treated with VB1 at various dosages, and the apoptosis was determined by flow cytometry with Annexin V and PI double staining. The cell lysates were prepared from RA cells treated with VB1 at various dosages, and western-blotting was performed by various antibodies as indicated. **e** RA cells were seeded into 6-well plates after being treated with VB1 at various dosages, and the distribution of the cell cycle was determined by flow cytometry as described in the Methods. The results represent the mean (n = 4) ± SD of each group, and an asterisk (*) indicates a significant difference using one-way ANOVA (*p* < 0.05)

V600E mutations in BRAF occur commonly in cutaneous melanoma, and its targeted inhibitors, including vemurafenib, have been administered by the FDA as therapy for advanced melanoma patients with BRAF mutations. However, most patients encounter BRAFi resistance after inhibitor treatment. Therefore, we examined the effects of VB1 on BRAFi-resistant cells. BRAFi-resistant A375 was generated by continual treatment with PLX4720 (2 μM) for more than 3 months to obtain a “resistant” cell line (labeled RA) (Fig. 5a). The results showed that VB1 dramatically inhibited cell viability in a dosage- and time-dependent manner and that the IC50 value of VB1 was approximately 5 μM (Fig. 5b). Furthermore, the growth of melanoma cell colonies in plates was sharply reduced by VB1 treatment (Fig. 5c). Next, we analyzed the apoptosis and cell cycle in RA cells by VB1. As shown in Fig. 5d, left panel, VB1 induced 18.95% apoptosis at 5 μM and 33.3% apoptosis at 20 μM. As a result, the cleavage of PARP, BAX and BCL2 was affected significantly by VB1 treatment (Fig. 5d, right panel). For the cell cycle analysis, VB1 treatment resulted in significant arrest at G2/M phase and decreased the distribution of cells in G0/G1 phase (Fig. 5e).

We then performed RNA-seq to investigate the molecular mechanism of VB1 in RA cells. As shown in Additional file 4: Figure S4A, we found that the differential expression genes were enriched in JAK-STAT, FOXO, TNF and cell cycle signaling pathways and particularly the cell cycle pathway, which is similar to the results of the non-BRAFi-resistant melanoma cell lines. We verified the key differential expression genes in RA cells, which showed that the *P21* (*CDKN1A*), *PUMA* (*BBC3*) and *GADD45A* expression levels increased significantly and that the *CDK1*, *CDK6*, *CYCE* (*CCNE*), *MCM6* and *CYCA* (*CCNA*) genes were significantly down-regulated (Additional file 4: Figure S4B).

**Fig. 6:**
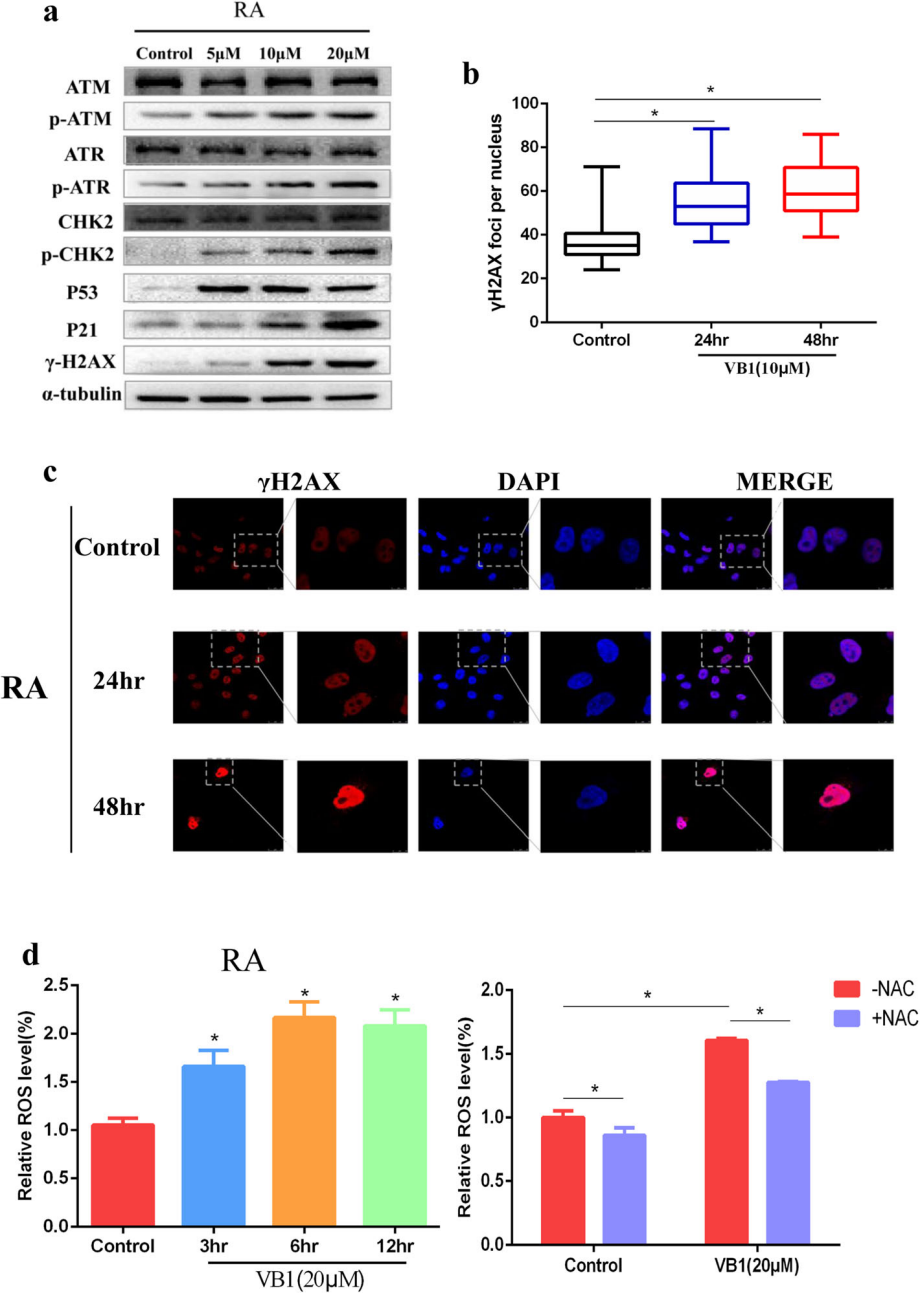
VB1 induces DNA damage by increasing ROS in RA cells. **a** RA cells were treated with 0–20 μM VB1 for 48 h, and western blotting was then performed for the indicated antibodies. **b** & **c** RA cells were treated with 10 μM VB1 for 0–48 h, and γH2AX was stained by immunofluorescence (**c**), the foci of γH2AX was quantificated (**b**), the results represent the mean (*n* = 5) ± SD of each group, and an asterisk (*) indicates a significant difference using one-way ANOVA (*p* < 0.05). **d** RA cells were treated with 20 μM VB1 for 0–12 h. The levels of ROS were measured by DCF fluorescence with flow cytometry. The relative ROS levels were analyzed by GraphPad Prism software (histogram) (left panel). RA cells were pretreated with 5 mmol/L N-acetylcysteine (NAC) for 1 h, and then exposed to 20 μM VB1 for another 6 h. The levels of ROS were measured by DCF fluorescence with flow cytometry. The relative ROS levels were analyzed using the GraphPad Prism software (histogram) (right panel). The results represent the mean (*n* = 4) ± SD of each group, and an asterisk (*) indicates a significant difference using one-way ANOVA (*p* < 0.05)

The previous results showed that VB1 could induce DNA damage through upregulation of the ROS level in melanoma cells. Therefore, we examined the effect of VB1 on DNA damage and the ROS level in BRAFi-resistant cells. The results indicated that VB1 increased P53 and the expression of its downstream molecule, P21, as well as P-ATM, P-ATR, P-CHK2 and γH2AX. There were no significant changes in the expression of total ATM, ATR and CHK2 protein level (Fig. 6a). In addition, we observed the accumulation of γH2AX in the nucleus of BRAFi-resistant cells (Fig. 6b, c). We also determined the intracellular ROS levels after VB1 treatment, which demonstrated that the role of VB1 in BRAFi-resistant cells was similar to that in nonresistant melanoma cell lines. Our results showed that VB1 treatment dramatically increases intracellular ROS levels in RA (Fig. 6d (left panel)), which could be partly impeded by antioxidant N-acetylcysteine (NAC) (Fig. 6d (right panel)). this indicates that VB1 could be used for the treatment of BRAFi-resistant melanoma patients.

## Discussion

In this study, we found that VB1 significantly blocks cell growth in various melanoma cell lines, including A375, Sk-Mel-28 and Sk-Mel-5, in vitro (Fig. 1b, d) and in vivo (Fig. 2d). Given the unbearable toxicity or side effects of chemotherapeutic drugs or targeted inhibitors that lead to failure in clinical treatments, we also examined the toxic effects of VB1. We treated immortalized nontumorigenic mouse skin epidermal JB6 and human skin keratinocyte (HaCat) cells with various concentrations of VB1, and the results showed that the IC50 values of VB1 for the two cell lines were over 80 μM (Fig. 1e), whereas the IC50 values in melanoma cells were 5.03 μM for A375 and 15.83 μM for SK-MEL-28 (Fig. 1c). These results indicate that the cytotoxicity of VB1 is selective to tumor cells. Our results are consistent with those of a previous study, which showed that VB1 was well tolerated in tumor-bearing mice with no significant differences in the body serum levels of alanine aminotransferase, aspartate aminotransferase, creatinine, white blood cell and erythrocyte counts between the VB1 treated group and the control group [20]. We also found that VB1 could cause apoptosis and induce cell cycle arrest in G2/M phase (Fig. 2a, b). In addition, the expression of cleaved PARP and Bax increased after VB1 treatment, whereas that of BCL2 decreased (Fig. 2c), which indicates that VB1 induces melanoma cell apoptosis.

Next, we performed RNA-seq to investigate the effect of VB1 on the signaling pathways. The p53 pathway, cell cycle pathway and apoptosis pathway were shown to be significantly altered after VB1 treatment (Fig. 3b). Furthermore, we verified key gene expression with mRNA levels after treatment with VB1 in melanoma cells, which indicated that the *P21*, *PUMA* and *GADD45A* expression was significantly upregulated and that the expression of *MCM6*, *CDK1*, *CDK6*, *CYCE* and *CYCA* was significantly downregulated (Fig. 3c, d). These genes are crucial mediators in the cell cycle, apoptosis and DNA damage [24, 25]. P21, which is also called *Cip1* or *CDKN1A*, inhibits several cyclin-dependent kinases and induces cell cycle arrest. *GADD45* can induce DNA damage and cell cycle arrest in the G2/M phase by directly regulating DNA nucleotide excision repair [26, 27]. PU*MA*, which is a downstream molecule of P53, can lead to apoptosis by inducing intracellular ROS and DNA damage [28, 29]. Therefore, these differentially expressed genes may provide a molecular mechanism for how VB1 treatment dramatically induces cell cycle arrest in G2/M phase in melanoma cells.

P53- and P53-related signaling pathways play crucial roles in tumorigenesis. P53 is an important DNA damage response (DDR) component, allowing to repair limited DNA damage through cell cycle arrest or to eliminate cells with severe DNA damage via apoptosis [30, 31]. A variety of DNA toxicity stresses could activate P53- and p53-related pathways and lead to transactivation of downstream target genes to regulate the cell cycle, apoptosis and DNA damage repair [32, 33]. Although inactivation mutations or allele deletions of P53 are common in human cancers, more than 80% of human melanomas express P53 with a wild-type (WT) sequence, and the induction of P53 expression could significantly inhibit melanoma cell growth [34]. Our results showed that VB1 could induce G2/M arrest and apoptosis, which indicates that this compound might cause DNA toxicity for cells. As expected, our findings showed that DNA damage-related proteins [24, 35, 36], including P-ATM, P-ATR, P-CHK2 and γH2AX as well as P53 and its downstream P21, were significantly increased after VB1 treatment (Fig. 4a), which suggests that VB1 inhibits melanoma cell growth through DNA damage that eventually leads to P53 pathway-related cell cycle arrest and apoptosis.

In general, DNA toxicity stresses involve IR, radiomimetic compounds, topo I/II inhibitors, UV, H_2_O_2_ and ROS [36]. Of these stresses, reactive oxygen species (ROS) constitute a group of highly reactive small molecules, including H_2_O_2_, superoxide (O^2−^) and hydroxyl radicals [37]. Although ROS facilitates the progression of cancer to some extent, the accumulation of ROS reaches a threshold and then causes cell death [23]. Therefore, increasing the ROS level to induce cancer cell death is a well-known anticancer strategy [38–40]. Evidence shows that pharmaceutical compounds extracted from plants, such as resveratrol, levistolide A and piperlongumine, induce apoptosis through DNA toxicity by the induction of intracellular ROS [40–42]. In this study, we measured the level of intracellular ROS in melanoma after VB1 treatment, and the results showed that VB1 significantly increases ROS levels, which indicates that the cells were under high oxidative stress. γH2AX is an indicator of DNA damage that accumulates in the nucleus and indicates the presence of DNA damage [43, 44]. In this study, we found that γH2AX increased in the nucleus after VB1 treatment in a time-dependent manner, which indicates that VB1 caused DNA damage, G2/M cycle arrest and apoptosis by increasing intracellular ROS (Fig. 6).

The administration of BRAF inhibitors benefits the OS and RFS of melanoma patients; however, after approximately 6 months of treatment, patients experience fatal drug resistance or the recurrence of metastases. The reactivation of MAPK signaling is involved in BRAFi resistance, such as bypass activation in RAF, MEK1/2 and NRAS [10, 45, 46]. In addition, EGFR-STAT3, CDK2 and AXL/AKT signaling pathways play critical roles in BRAFi resistance in melanoma [12, 47, 48]. Interestingly, we found that VB1 could also significantly inhibit the growth of RA. The IC50 values are similar to those of its parental cells (approximately 3–5 μM), which indicates that VB1 could completely overcome the BRAFi resistance of melanoma. Similar to nonresistant melanoma cell lines, VB1 also increases intracellular ROS levels and induces DNA damage, leading to melanoma cell cycle arrest and apoptosis (Figs. 5, 6), which suggests that VB1 is applicable to BRAFi-resistant melanoma patients.

## Conclusions

**Fig. 7:**
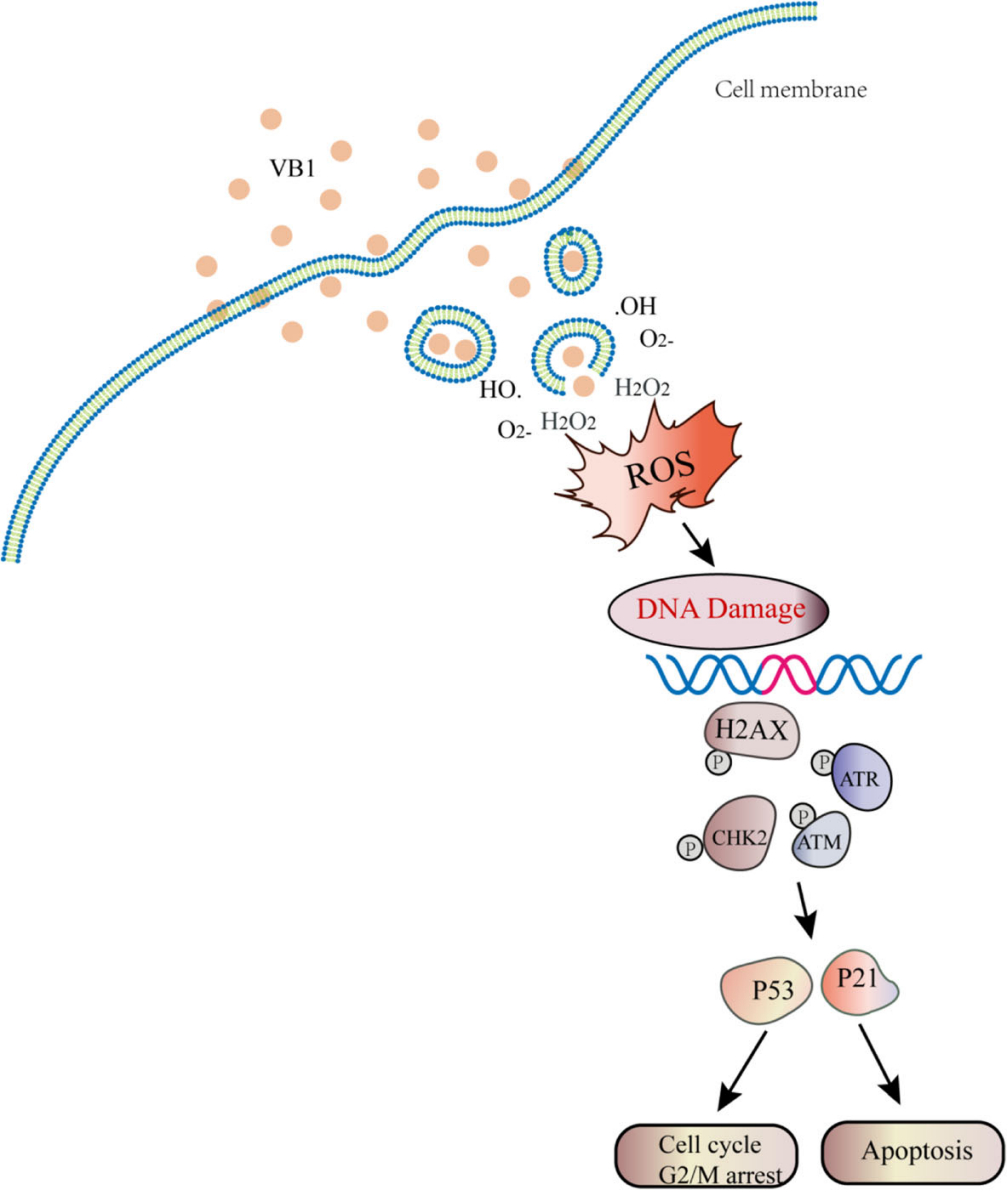
Schematic diagram of the mechanism of action of VB1. VB1 kills cancer cells by accumulating intracellular ROS, which leads to DNA damage, G2/M cell cycle arrest and apoptosis in melanoma cells

In summary, we demonstrated that VB1 promotes the accumulation of intracellular ROS, resulting in DNA damage, G2/M cell cycle arrest and apoptosis in melanoma cells and BRAFi-resistant melanoma cells, which provides evidence for the application of VB1 as an antimelanoma treatment (Fig. 7).

## Additional files

Additional file 1: **Figure S1.** VB1 inhibits melanoma cells growth in vitro and in vivo. (A) Sk-Mel-5 cells were prepared in 96-well plates. The cells were treated with VB1 for various times and dosages as indicated, and cell viability was tested by CCK-8 as described in the *Materials and Methods*. The results represent the mean (*n*=6) ± SD of each group, and an asterisk (*) indicates a significant difference using one-way ANOVA (*p* < 0.05). The IC50 values of VB1 in Sk-Mel-5 were automatically calculated by GraphPad Prism software as described in the Materials and Methods (lower panel). (B) Sk-Mel-5 cells were seeded into 6-well plates and then treated with various dosages of VB1 as indicated for 24 h. After 10–14 days, the number of colonies was assessed and quantified by crystal violet staining as described in the *Materials and Methods*. (C) Sk-Mel-5 cells were treated with various dosages of VB1 for 48 h, and the extent of apoptosis was determined by flow cytometry with Annexin V and PI double staining as described in the *Materials and Methods*. (D) Sk-Mel-5 cells treated with various dosages of VB1 were lysed, and western blotting was then performed for the indicated antibodies. (E) Sk-Mel-5 cells were treated with various dosages of VB1 for 48 h. The cell cycle distribution was detected by flow cytometry as described in the *Materials and Methods*. The results represent the mean (*n*=4) ± SD of each group, and an asterisk (*) indicates a significant difference using one-way ANOVA (*p* < 0.05). (F) Sk-Mel-5 melanoma cells (2×10^6^ cells/0.15 mL) were xenografted into nude mice. When the tumors reached approximately 50 mm^3^, the tumor-bearing mice were randomized for intraperitoneal injection of 40 or 80 mg/kg of VB1 twice every other day for 2–3 weeks as described in the *Materials and Methods*. The overview of nude mice were showed. (G) The tumor body weight were measured twice per week. The results are shown as the mean tumor volume ± SD, and an asterisk (*) indicates a significant difference (*p* < 0.05 one-way ANOVA). (TIF 6050 kb)

Additional file 2: **Figure S2.** RNA-seq analyses of the effect of VB1 on the gene expression profile in SK-MEL-5 cells. (A) SK-MEL-5 cells were treated with 10 µM VB1 for 48 h. RNA-seq was performed as described in the *Materials and Methods*, and differential expression genes were analyzed using DESeq2. The KEGG pathway was used to analyze the pathways related to the differential expression genes. The top 20 positively enriched pathways are shown in the bubble chart. The *x*-axis is the enrichment score, and the *y*-axis is the enriched pathways. (B) RNA was extracted from Sk-Mel-5 treated with VB1 as indicated, and RT-Q-PCR was then performed as described in the *Materials and Methods*. The data from multiple experiments are expressed as the mean ± S.D. Significant differences were evaluated using one-way ANOVA, and an asterisk (*) indicates a significant difference (*p* < 0.05). (TIF 3060 kb)

Additional file 3: **Figure S3.** VB1 treatment induces DNA damage by increasing ROS in vitro and in vivo. (A) Sk-Mel-5 cells were treated with 0–20 µM VB1 for 48 h, and western blotting was then performed for the indicated antibodies. (B) Sk-Mel-5 cells were treated with 10 µM VB1 for 0–48 h, and γH2AX was stained by immunofluorescence and calculated. The results represent the mean (*n*=5) ± SD of each group, and an asterisk (*) indicates a significant difference using one-way ANOVA (*p* < 0.05). (C) Sk-Mel-5 cells were treated with 10 µM VB1 for 0–48 h, and γH2AX was stained by immunofluorescence. Representative images of staining of γH2AX. (D) Sk-Mel-5 cells were treated with 20 µM VB1 for 0–12 h. The levels of ROS were measured by DCF fluorescence with flow cytometry. The relative ROS levels were analyzed using the GraphPad Prism software (histogram) (left panel). Sk-Mel-5 cells were pretreated with 5 mmol/L N-acetylcysteine (NAC) for 1 h, and then, exposed to 20µM VB1 for another 6 h. The levels of ROS were measured by DCF fluorescence with flow cytometry. The relative ROS levels were analyzed using the GraphPad Prism software (histogram) (right panel). The results represent the mean (*n*=4) ± SD of each group, and an asterisk (*) indicates a significant difference using one-way ANOVA (*p* < 0.05). (D) The immunohistochemistry was performed as indicated antibodies as described in the *Materials and Methods*. (TIF 8570 kb)

Additional file 4: **Figure S4.** RNA-seq analyses of the effect of VB1 on the gene expression profile in RA cells (A) RA cells were treated with 10 µM VB1 for 48 h. RNA-seq was performed as described in the Materials and Methods, and differential expression genes were analyzed using DESeq2. The KEGG pathway was used to analyze the pathways related to the differential expression genes. The top 20 positively enriched pathways are shown in the bubble chart. The *x*-axis is the enrichment score, and the *y*-axis is the enriched pathways. (B) RNA was extracted from RA treated with VB1 as indicated, and RT-Q-PCR was then performed as described in the Materials and Methods. The data from multiple experiments are expressed as the mean ± S.D. Significant differences were evaluated using one-way ANOVA, and an asterisk (*) indicates a significant difference (*p* < 0.05). (TIF 3770 kb)

## Abbreviations

BRAFi: BRAF inhibitor
CCK8: Cell counting kit
DCFH-DA: 2,7-Dichlorodi -hydrofluorescein diacetate
DMEM: Dulbecco’s modified Eagle’s medium
DMSO: Dimethyl sulfoxide
EDTA: Ethylene Diamine Tetraacetic Acid
FBS: Fatal bovine serum
FDA: Food and Drug Administration
IC50: half maximal inhibitory concentration
MEKi: Mitogen-activated protein kinase kinase inhibitor
PBS: Phosphate Buffered Saline
RIPA: Radio-Immunoprecipitation Assay
ROS: Reactive oxygen species
SDS-PAGE: SDS-polyacrylamide gel electrophoresis
VB1: Vitexin compound 1
