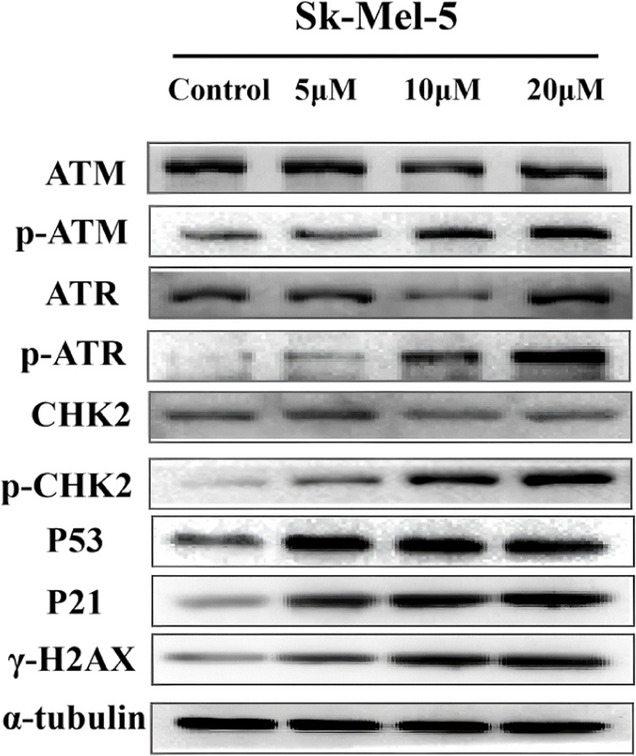# Correction: Vitexin compound 1, a novel extraction from a Chinese herb, suppresses melanoma cell growth through DNA damage by increasing ROS levels

**DOI:** 10.1186/s13046-026-03761-z

**Published:** 2026-06-22

**Authors:** Nian Liu, Kuan Song Wang, Min Qi, Ying Jun Zhou, Guang Yao Zeng, Juan Tao, Jian Da Zhou, Jiang Lin Zhang, Xiang Chen, Cong Peng

**Affiliations:** 1 Department of Dermatology, Xiangya Hospital, Central South University, Changsha, Hunan China; 2 Hunan Key Laboratory of Skin Cancer and Psoriasis, Changsha, Hunan China; 3 Department of Pathology, Xiangya Hospital, Central South University, Changsha, Hunan China; 4 Department of Pathology, School of Basic Medical Sciences, Central South University, Changsha, Hunan China; 5 Department of Plastic and Cosmetic Surgery, XiangYa Hospital, Central South University, Changsha, Hunan China; 6 School of Pharmaceutical Science, Central, South University, Changsha, Hunan China; 7 Department of Dermatology, Affiliated Union Hospital, Tongji Medical College, Huazhong University of Science and Technology, Wuhan, China; 8 Department of Plastic Surgery of Third Xiangya Hospital, Central South University, Changsha, China

**Correction: J Exp Clin Cancer Res 37, 269 (2018)**

**https://doi.org/10.1186/s13046-018-0897-x**

Following publication of the original article [1], the authors spotted errors in the right panel of Figure 4a. Errors were also spotted in the Supplementary Figure S3a.

The correct figures are presented below:

Incorrect Fig. 4a (right panel)

**Fig. 4:**
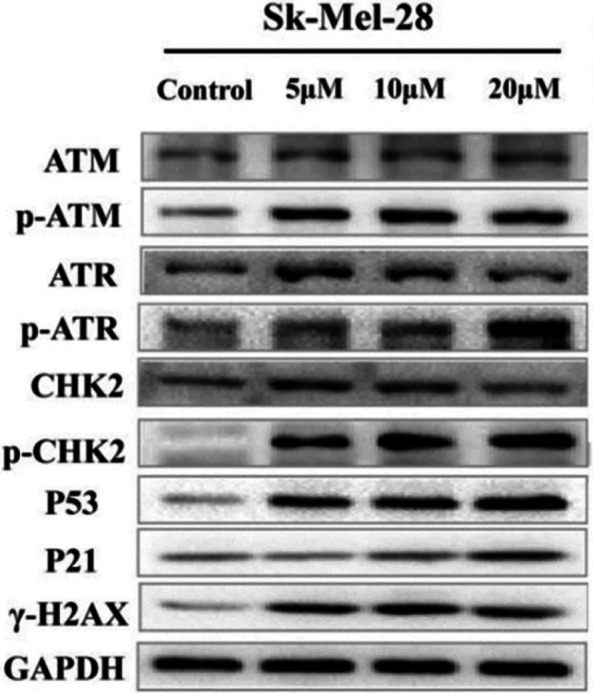
VB1 treatment induces DNA damage by increasing ROS. **a** A375 (left panel) and Sk-Mel-28 (right panel) cells were treated with 0–20 μM VB1 for 48 h, and western blotting was then performed for the indicated antibodies. **b** A375 and Sk-Mel-28 cells were treated with 10 μM VB1 for 0–48 h, and γH2AX was stained by immunofluorescence and calculated. The results represent the mean (*n* = 5) ± SD of each group, and an asterisk (*) indicates a significant difference using one-way ANOVA (*p* < 0.05). **c** A375 and Sk-Mel-28 cells were treated with 10 μM VB1 for 0–48 h, and γH2AX was stained by immunofluorescence. Representative images of staining of γH2AX. **d** A375 and Sk-Mel-28 cells were treated with 20 μM VB1 for 0–12 h. The levels of ROS were measured by DCF fluorescence with flow cytometry. The relative ROS levels were analyzed using the GraphPad Prism software (histogram). **e** A375 and Sk-Mel-28 cells were pretreated with 5 mmol/L N-acetylcysteine (NAC) for 1 h, and then, exposed to 20 μM VB1 for another 6 h. The levels of ROS were measured by DCF fluorescence with flow cytometry. The relative ROS levels were analyzed using the GraphPad Prism software (histogram). The results represent the mean (*n* = 4) ± SD of each group, and an asterisk (*) indicates a significant difference using one-way ANOVA (*p* < 0.05)

Correct Fig. 4a (right panel)

**Fig. 4:**
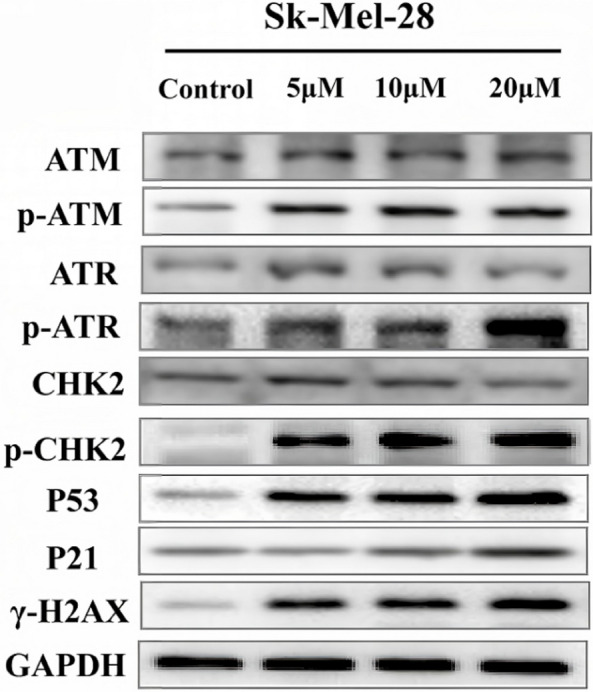
VB1 treatment induces DNA damage by increasing ROS. **a** A375 (left panel) and Sk-Mel-28 (right panel) cells were treated with 0–20 μM VB1 for 48 h, and western blotting was then performed for the indicated antibodies. **b** A375 and Sk-Mel-28 cells were treated with 10 μM VB1 for 0–48 h, and γH2AX was stained by immunofluorescence and calculated. The results represent the mean (*n* = 5) ± SD of each group, and an asterisk (*) indicates a significant difference using one-way ANOVA (*p* < 0.05). **c** A375 and Sk-Mel-28 cells were treated with 10 μM VB1 for 0–48 h, and γH2AX was stained by immunofluorescence. Representative images of staining of γH2AX. **d** A375 and Sk-Mel-28 cells were treated with 20 μM VB1 for 0–12 h. The levels of ROS were measured by DCF fluorescence with flow cytometry. The relative ROS levels were analyzed using the GraphPad Prism software (histogram). **e** A375 and Sk-Mel-28 cells were pretreated with 5 mmol/L N-acetylcysteine (NAC) for 1 h, and then, exposed to 20 μM VB1 for another 6 h. The levels of ROS were measured by DCF fluorescence with flow cytometry. The relative ROS levels were analyzed using the GraphPad Prism software (histogram). The results represent the mean (*n* = 4) ± SD of each group, and an asterisk (*) indicates a significant difference using one-way ANOVA (*p* < 0.05)

**Incorrect Fig. S3a** 
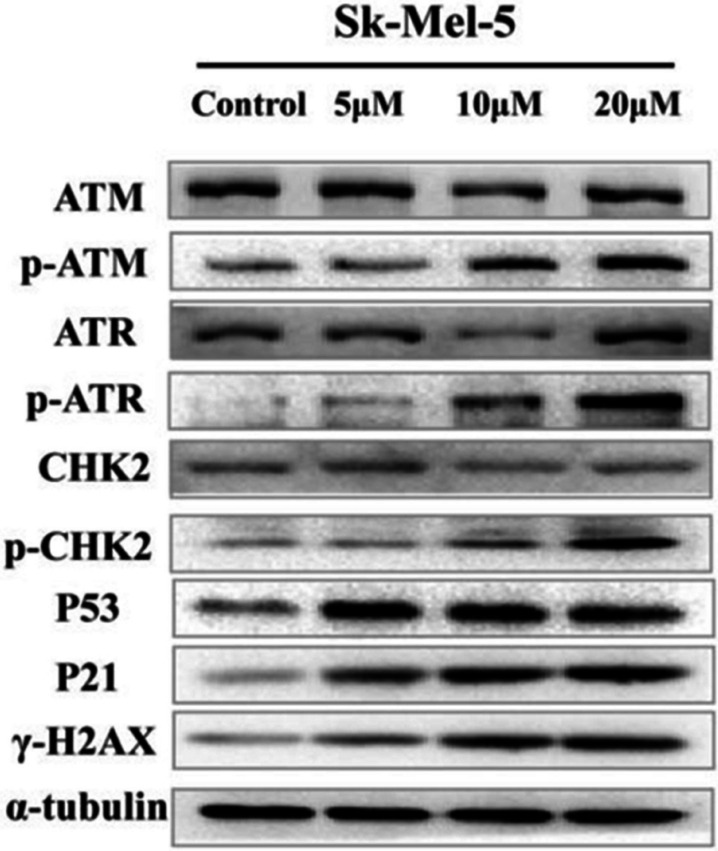


**Correct Fig. S3a**