# Supplementary figures and images for: Vitexin compound 1, a novel extraction from a Chinese herb, suppresses melanoma cell growth through DNA damage by increasing ROS levels
Source: J Exp Clin Cancer Res. 2018 Nov 6;37:269. doi: 10.1186/s13046-018-0897-x (PMC6219156; doi:10.1186/s13046-018-0897-x)

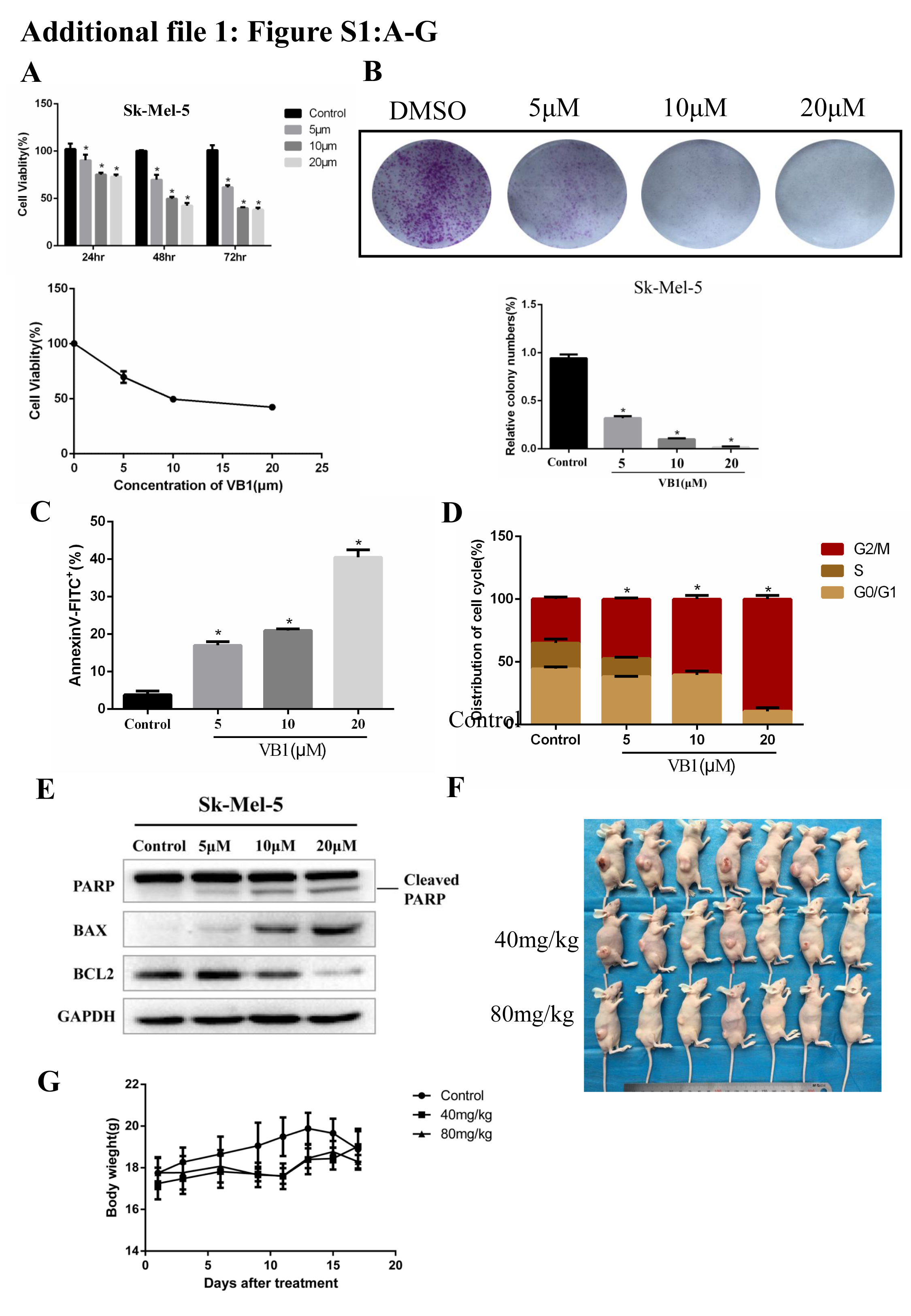

Supplement: Supplementary file 1 — Figure S1. VB1 inhibits melanoma cells growth in vitro and in vivo. (A) Sk-Mel-5 cells were prepared in 96-well plates. The cells were treated with VB1 for various times and dosages as indicated, and cell viability was tested by CCK-8 as described in the Materials and Methods. The results represent the mean (n=6) ± SD of each group, and an asterisk (*) indicates a significant difference using one-way ANOVA (p < 0.05). The IC50 values of VB1 in Sk-Mel-5 were automatically calculated by GraphPad Prism software as described in the Materials and Methods (lower panel). (B) Sk-Mel-5 cells were seeded into 6-well plates and then treated with various dosages of VB1 as indicated for 24 h. After 10–14 days, the number of colonies was assessed and quantified by crystal violet staining as described in the Materials and Methods. (C) Sk-Mel-5 cells were treated with various dosages of VB1 for 48 h, and the extent of apoptosis was determined by flow cytometry with Annexin V and PI double staining as described in the Materials and Methods. (D) Sk-Mel-5 cells treated with various dosages of VB1 were lysed, and western blotting was then performed for the indicated antibodies. (E) Sk-Mel-5 cells were treated with various dosages of VB1 for 48 h. The cell cycle distribution was detected by flow cytometry as described in the Materials and Methods. The results represent the mean (n=4) ± SD of each group, and an asterisk (*) indicates a significant difference using one-way ANOVA (p < 0.05). (F) Sk-Mel-5 melanoma cells (2×10^6^ cells/0.15 mL) were xenografted into nude mice. When the tumors reached approximately 50 mm3, the tumor-bearing mice were randomized for intraperitoneal injection of 40 or 80 mg/kg of VB1 twice every other day for 2–3 weeks as described in the Materials and Methods. The overview of nude mice were showed. (G) The tumor body weight were measured twice per week. The results are shown as the mean tumor volume ± SD, and an asterisk (*) indicates a significant difference (p [file 13046_2018_897_MOESM1_ESM.tif]

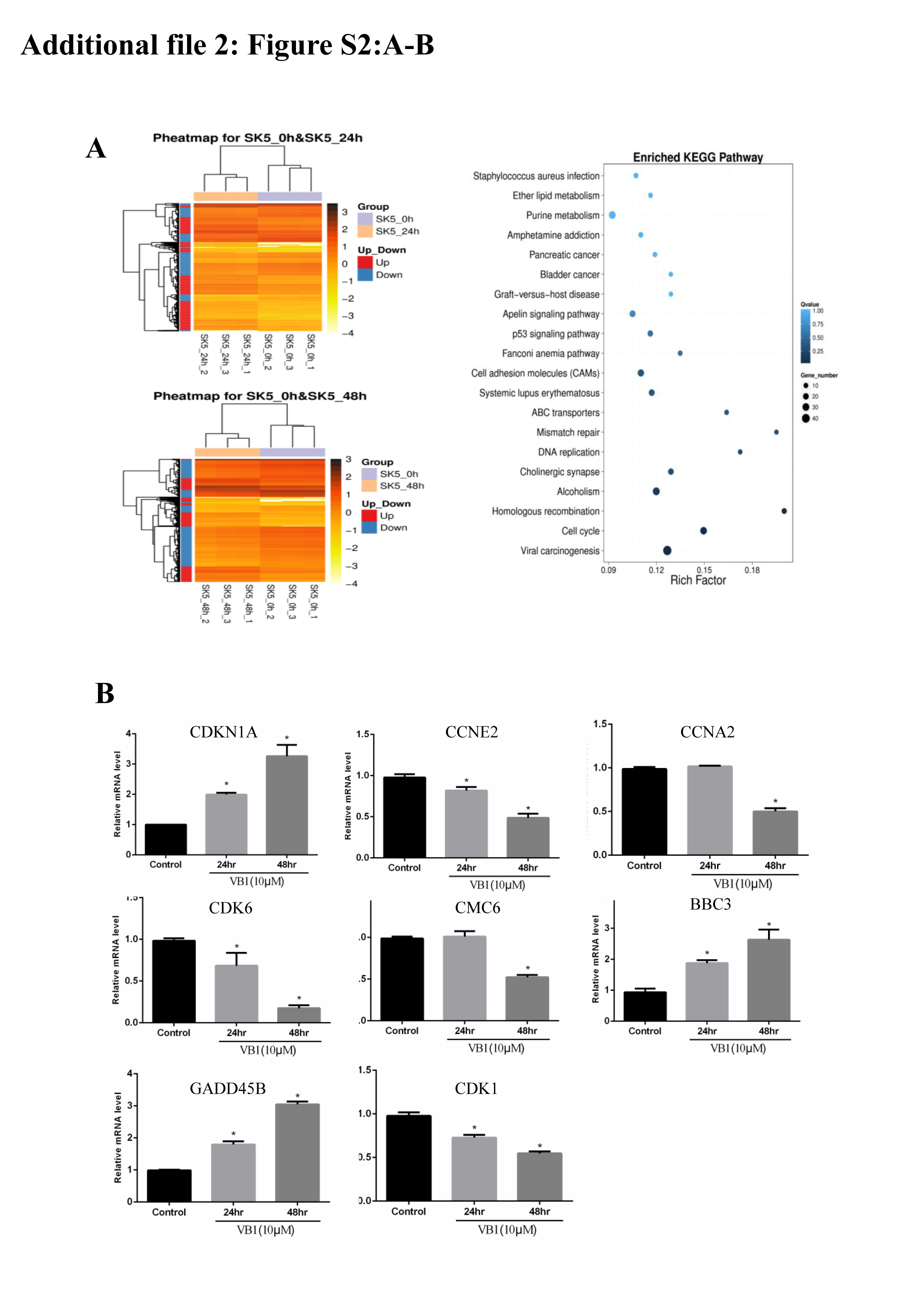

Supplement: Supplementary file 2 — Figure S2. RNA-seq analyses of the effect of VB1 on the gene expression profile in SK-MEL-5 cells. (A) SK-MEL-5 cells were treated with 10 µM VB1 for 48 h. RNA-seq was performed as described in the Materials and Methods, and differential expression genes were analyzed using DESeq2. The KEGG pathway was used to analyze the pathways related to the differential expression genes. The top 20 positively enriched pathways are shown in the bubble chart. The x-axis is the enrichment score, and the y-axis is the enriched pathways. (B) RNA was extracted from Sk-Mel-5 treated with VB1 as indicated, and RT-Q-PCR was then performed as described in the Materials and Methods. The data from multiple experiments are expressed as the mean ± S.D. Significant differences were evaluated using one-way ANOVA, and an asterisk (*) indicates a significant difference (p < 0.05). (TIF 3060 kb) [file 13046_2018_897_MOESM2_ESM.tif]

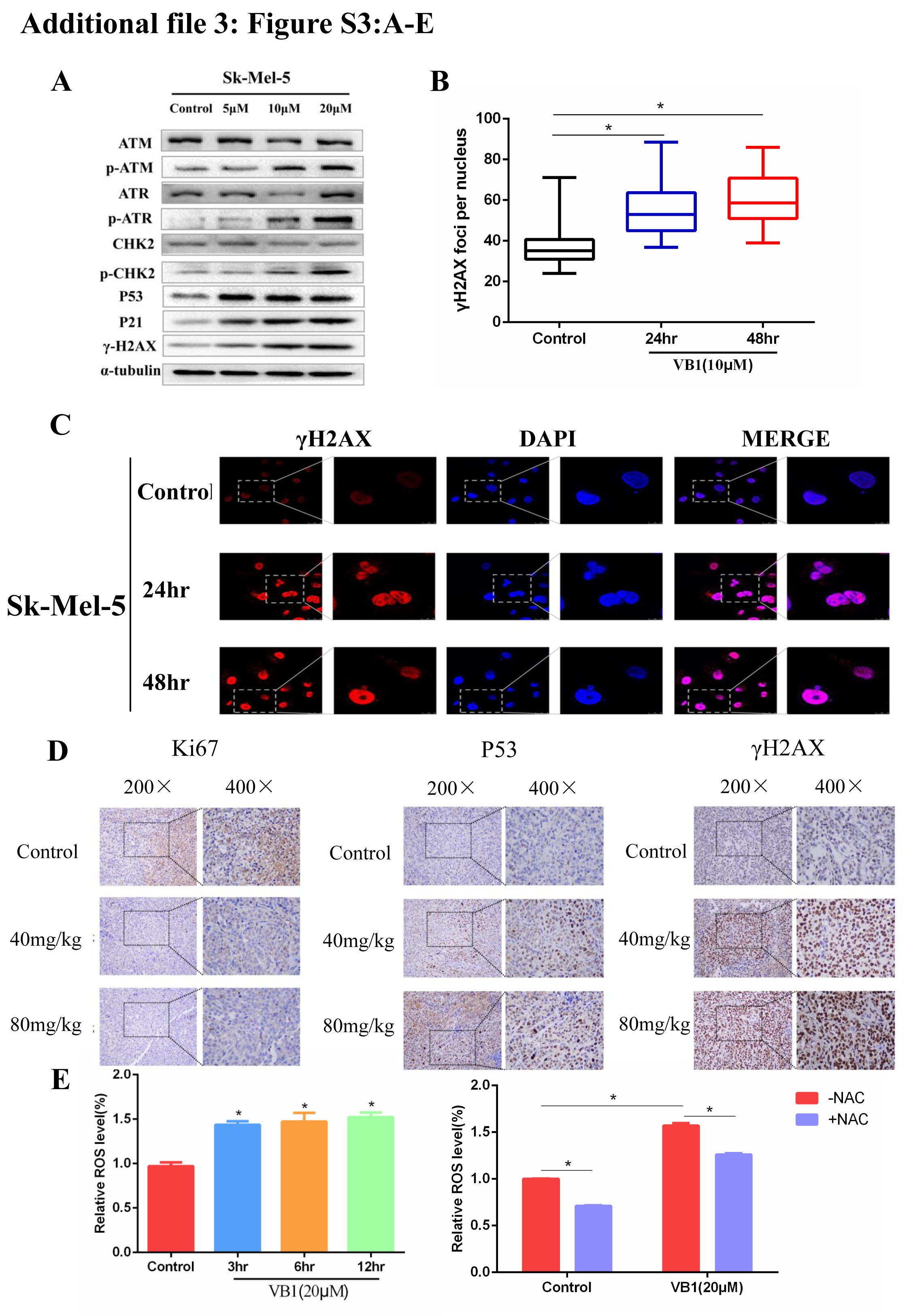

Supplement: Supplementary file 3 — Figure S3. VB1 treatment induces DNA damage by increasing ROS in vitro and in vivo. (A) Sk-Mel-5 cells were treated with 0–20 µM VB1 for 48 h, and western blotting was then performed for the indicated antibodies. (B) Sk-Mel-5 cells were treated with 10 µM VB1 for 0–48 h, and γH2AX was stained by immunofluorescence and calculated. The results represent the mean (n=5) ± SD of each group, and an asterisk (*) indicates a significant difference using one-way ANOVA (p < 0.05). (C) Sk-Mel-5 cells were treated with 10 µM VB1 for 0–48 h, and γH2AX was stained by immunofluorescence. Representative images of staining of γH2AX. (D) Sk-Mel-5 cells were treated with 20 µM VB1 for 0–12 h. The levels of ROS were measured by DCF fluorescence with flow cytometry. The relative ROS levels were analyzed using the GraphPad Prism software (histogram) (left panel). Sk-Mel-5 cells were pretreated with 5 mmol/L N-acetylcysteine (NAC) for 1 h, and then, exposed to 20µM VB1 for another 6 h. The levels of ROS were measured by DCF fluorescence with flow cytometry. The relative ROS levels were analyzed using the GraphPad Prism software (histogram) (right panel). The results represent the mean (n=4) ± SD of each group, and an asterisk (*) indicates a significant difference using one-way ANOVA (p < 0.05). (D) The immunohistochemistry was performed as indicated antibodies as described in the Materials and Methods. (TIF 8570 kb) [file 13046_2018_897_MOESM3_ESM.tif]

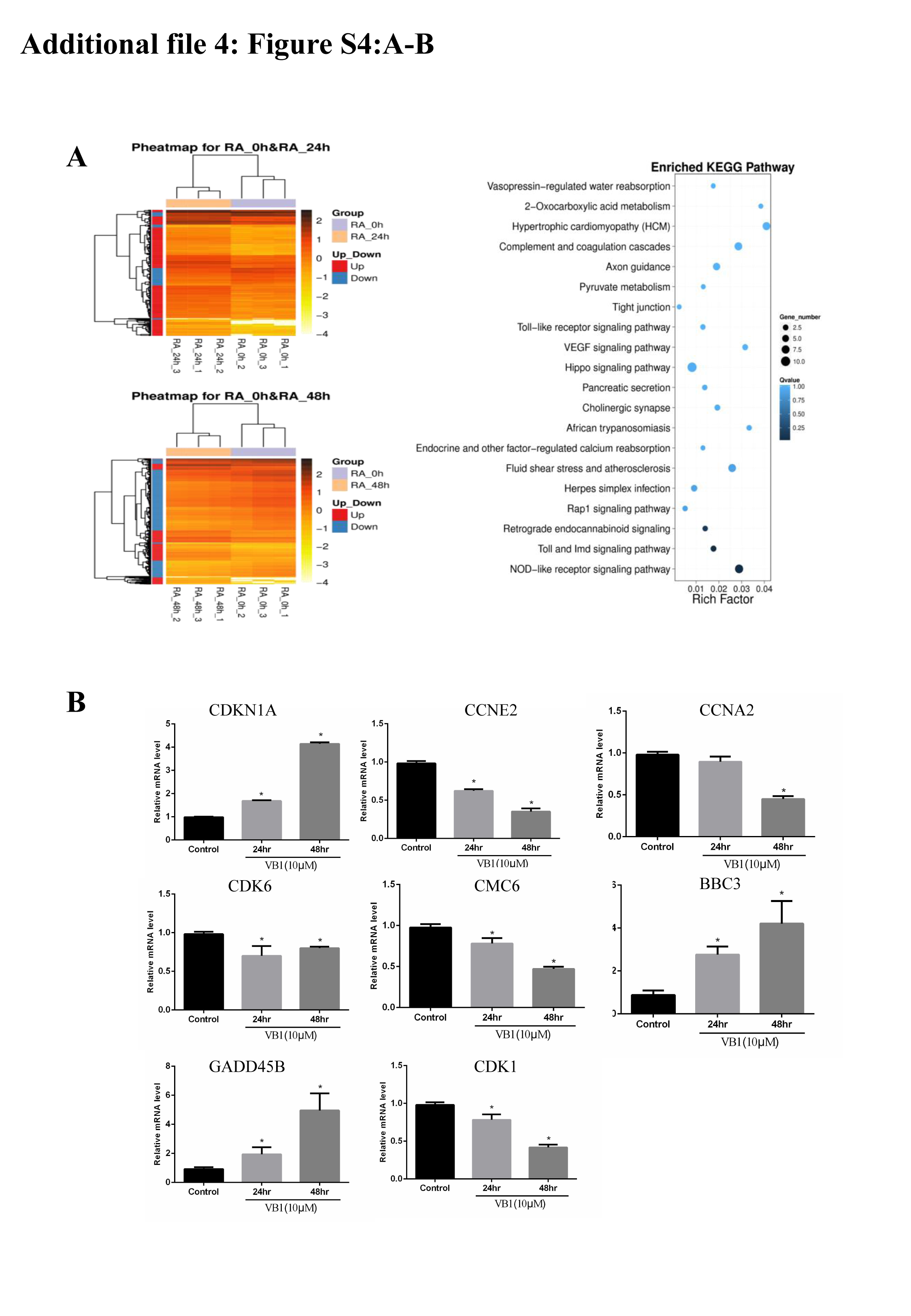

Supplement: Supplementary file 4 — Figure S4. RNA-seq analyses of the effect of VB1 on the gene expression profile in RA cells (A) RA cells were treated with 10 µM VB1 for 48 h. RNA-seq was performed as described in the Materials and Methods, and differential expression genes were analyzed using DESeq2. The KEGG pathway was used to analyze the pathways related to the differential expression genes. The top 20 positively enriched pathways are shown in the bubble chart. The x-axis is the enrichment score, and the y-axis is the enriched pathways. (B) RNA was extracted from RA treated with VB1 as indicated, and RT-Q-PCR was then performed as described in the Materials and Methods. The data from multiple experiments are expressed as the mean ± S.D. Significant differences were evaluated using one-way ANOVA, and an asterisk (*) indicates a significant difference (p < 0.05). (TIF 3770 kb) [file 13046_2018_897_MOESM4_ESM.tif]
